# Frailty in primary care: challenges, innovations, and future directions

**DOI:** 10.1186/s12875-023-02083-9

**Published:** 2023-06-23

**Authors:** Kristiana Ludlow, Oliver Todd, Natasha Reid, Hakan Yaman

**Affiliations:** 1 Centre for Health Services Research, Faculty of Medicine, the University of Queensland, Brisbane, Australia; 2 Academic Unit for Ageing and Stroke Research, University of Leeds, Bradford Teaching Hospitals, Bradford, UK; 3 Anatolia Hospital, Antalya, Türkiye

**Keywords:** Frailty, Primary Care, Older adults

## Abstract

Frailty is one of the biggest challenges to healthy ageing, and yet our understanding and management of frailty is in its infancy. In this editorial we outline challenges, innovations and future directions in frailty research in primary care, and invite contributions to *BMC Primary Care*’s “Frailty in Primary Care” Collection.

## Main body

In an ageing society, understanding how to maintain good health in old age presents one of the defining challenges of our times. The negative consequences of ageing have been characterised as frailty; however, frailty is not inevitable, and there has been a growing body of research over the past 20 years demonstrating new insights into frailty prevention and management to improve the health of older adults. *BMC Primary Care* welcomes submissions to its new “Frailty in Primary Care” Collection to help further our understanding of identifying, measuring, preventing and managing frailty, and supporting those who are living with frailty.

Frailty refers to a loss of physiological reserve and a failure of homeostasis to maintain a steady state in the face of stressors (e.g., an infection, fall, new medication or environmental change). These stressors can cause dramatic changes in the person—from being lucid to delirious, mobile to being bedbound, independent to requiring care for basic daily needs [1].

Frailty operates on a spectrum and is characterised by an increased risk of poor outcomes, including longer and more frequent hospitalisations, more peri-operative complications, greater functional needs, long-term disability, and mortality [2]. Prevalence estimates of frailty in older community-dwelling adults range from 4 to 59%, with an overall weighted average frailty prevalence of 10.7% and a pre-frailty prevalence of 41.6% [3]. Groups disproportionally affected by frailty include women [3], people living with socioeconomic deprivation [3], those living in long-term care facilities [4], and people living with severe mental illness [5].

Primary care providers are in a unique position to identify and manage frailty as they can intervene early in a patient’s frailty trajectory. They may have the opportunity to develop ongoing relationships with patients over time, affording them a more comprehensive understanding of patients’ health status, medical history, and social circumstances. As such, primary care providers can develop individualised care plans to address physical activity, social and emotional needs, nutritional status, and optimisation of medication.

We are in the infancy of understanding and measuring frailty. We know that over time, humans accumulate damage at a cellular and molecular level. These changes include genomic instability, telomere attrition, epigenetic alterations, mitochondrial dysfunction, and cellular senescence [6]. The onset and rate of damage, and the degree to which damage is repaired varies between individuals, and relates to factors such as smoking, exercise, obesity and deprivation across the life course.

There is no consensus on the best way to measure frailty. A recent systematic review found at least 60 tools for identifying frailty, nine of which are in regular use [7]. Frailty tools are validated for use in particular contexts. Choosing which measurement of frailty is appropriate will depend on several factors, including the purpose (e.g., population screening vs. individual treatment), setting (e.g., primary care, community, hospital) and who is making the measurement (the patient, caregiver, or clinician).

Evidence suggests that health outcomes may be improved for people with frailty through multi-component interventions, preventative screening, and frailty assessment. A recent network meta-analysis of 69 randomised control trials demonstrated that physical activity was the most effective intervention in reducing frailty, specifically resistance training, but the certainty of the available evidence was low-moderate [8].

There is also strong evidence that the Comprehensive Geriatric Assessment (CGA) reduces adverse outcomes associated with frailty [9], particularly in the tertiary setting. CGA provides a multidimensional and comprehensive assessment of an older person’s physical, social, psychological and cognitive status that informs an individualised care plan to improve health and wellbeing. However, CGA has been variously defined in the literature, there is insufficient guidance about which are its most effective components, and more evidence is required in the primary health setting. The World Health Organization’s Age-Friendly PHC Centres Toolkit offers another avenue for primary care workers to assess and manage older adults’ health. However, this tool does not make explicit reference to frailty. In primary care settings, the use of tools may be hindered by time constraints. Routinely collected data offers a means to identify older people living with frailty (e.g., electronic frailty index [10]), for example, to offer targeted interventions such as personalised care planning. However, frailty-relevant routinely collected data is often lacking in primary care, particularly in low- and middle-income countries. Efforts should be made to include frailty-specific items to minimum primary healthcare datasets, without overwhelming the daily routines of staff.

Another gap in clinical practice is the need for education and training for healthcare professionals. Many healthcare professionals lack awareness about frailty including its screening, assessment, and management. A systematic review including nine programs evaluating healthcare training in frailty found that they increased healthcare professionals’ knowledge of frailty and self-reported competence in assessing frailty [11]. Interdisciplinary management of frailty is necessary, for example, close partnerships between primary care, rehabilitation, mental health, and allied health. In countries with limited resources, there is a need for alternative pathways to ensure frail people have barrier-free access to optimal frailty management in the community and at home.

There is a need to establish evidence-based practices, pathways and interventions targeting frailty in different disease settings, including primary care, particularly in rural and remote communities. Examples include growing evidence to support the management of hypertension in the context of frailty [12], consensus guidelines for the management of diabetes according to frailty [13] and the Asia Pacific Clinical Frailty Guidelines [14].

Across frailty research, partnering with older people and caregivers is crucial. Older people can sometimes be reluctant to acknowledge their frailty, as frailty tends to be viewed as a negative and value-laden term [15]. It is important to meaningfully partner with older people and their supports/representatives to understand how to better design care to meet the older person’s needs and improve outcomes relevant to older people.

We invite submissions to *BMC Primary Care*’s “Frailty in Primary Care” Collection. We hope this collection will shine a spotlight on exciting new developments in frailty research which focuses on the prevention and management of frailty and further advances the growing evidence base for the care of older people in primary care settings.

## Data Availability

Not applicable.

## Abbreviation

CGA: Comprehensive Geriatric Assessment
